# Comparison of global and regional myocardial strains in patients with heart failure with a preserved ejection fraction vs hypertension vs age-matched control

**DOI:** 10.1186/s12947-020-00223-0

**Published:** 2020-11-10

**Authors:** Hyung Yoon Kim, Sung-Ji Park, Sang-Chol Lee, Shin Yi Chang, Eun-Kyoung Kim, Sung-A Chang, Jin-Oh Choi, Seung Woo Park, Sung-Mok Kim, Yeon Hyeon Choe, Jae K. Oh

**Affiliations:** 1 Division of Cardiology, Cardiovascular Imaging Center, Department of Internal Medicine, Heart Vascular Stroke Institute, Samsung Medical Center, Sungkyunkwan University School of Medicine, 81 Irwon-ro, Gangnam-gu, Seoul, 06351 Republic of Korea; 2 Present Address: Department of Cardiovascular medicine, Chonnam National University Medical school/Hospital, Gwangju, Republic of Korea; 3 Department of Radiology, Cardiovascular Imaging Center, Heart Vascular Stroke Institute, Samsung Medical Center, Sungkyunkwan University School of Medicine, Seoul, Republic of Korea; 4 Division of Cardiovascular Diseases, Department of Internal Medicine, Mayo Clinic, Rochester, MN USA

**Keywords:** Global strain, Regional strain, HFpEF, Hypertension, Speckle-tracking echocardiography

## Abstract

**Background:**

With an increasing clinical importance of the treatment of the heart failure (HF) with preserved ejection fraction (HFpEF), it is important to be certain of the diagnosis of HF. We investigated global and regional left ventricular (LV) strains using speckle tracking echocardiography (STE) in patients with HFpEF and compared those parameters with that of patients with hypertension and normal subjects.

**Methods:**

Peak longitudinal, circumferential and radial strains were assessed globally and regionally for each study groups using STE. Diastolic strain rate was also determined.

**Results:**

There were 50 patients in HFpEF group, 56 patients in hypertension group and 46 age-matched normal subjects. In patients with HFpEF, global peak longitudinal, circumferential and radial strain and strain rate were reduced compared to both hypertension patients and normal controls (− 15.5 ± 5.3 vs − 17.7 ± 3.1 and − 19.9 ± 2.0; − 9.7 ± 2.2 vs − 19.3 ± 3.1 and − 20.5 ± 3.3; 17.7 ± 8.2 vs 38.4 ± 12.4 and 43.6 ± 11.9, respectively, *P* <  0.001, for all). The diagnostic performance of global circumferential strain to predict the HFpEF was greatest among strain parameters (area under the curve = 0.997).

**Conclusions:**

In the speckle tracking echocardiography, impaired peak global strain and homogeneously reduced regional strain was observed in HFpEF patients compared to the hypertension patients and normal subjects in decreasing order. This can provide early information on the initiation of LV deformation of HFpEF in patients with hypertension or normal subjects.

## Background

Clinical importance of the diagnosis and the treatment of the heart failure with preserved ejection fraction (HFpEF) became significant, since HFpEF is accounted for half of the entire heart failure (HF) population and its clinical outcomes are similar to that of heart failure with reduced ejection fraction (HFrEF) [1].

However, non-invasive diagnosis of HFpEF is challenging because HFpEF encompasses various pathophysiological background and its clinical characteristics. Moreover, the definition of HFpEF with left ventricular (LV) systolic function by conventional echocardiography has been changed several times over time.

Two dimensional speckle tracking echocardiography (2D-STE) enables to detect subclinical LV dysfunction in the earlier phase of disease and to differentiate various degrees of the subclinical LV dysfunction [2, 3].

Similarly, LV myocardial strain is reduced in hypertensive subject compared to normal subject regardless of presence of LV hypertrophy [4]. Hypertension is considered to be a dominant risk factor of the HFpEF. An incidence of the HF among hypertension subjects is reported to be 1 ~ 2% per year [5].

Identifying subclinical LV systolic dysfunction among hypertensive subjects might be helpful in differentiate patients at higher risk for the development of HF. However, it is challenging to distinguish HFpEF and hypertensive heart disease, because they have similar cardiovascular features including symptom and LV ejection fraction (EF) on conventional echocardiography.

Accordingly, we hypothesized that earlier detection of LV dysfunction in patients with hypertension can be accomplished by quantifying myocardial strain using 2D-STE. The objectives of this study were to analyze global and regional LV strains using 2D-STE in patients with HFpEF and to compare those parameters with that of patients with hypertension and that of normal subjects.

## Methods

### Study design and population

This study is a prospective, multicenter international cardiac imaging study of HF (IMAGING-HF study). From March 2009 to March 2011, patients with HF were enrolled in this study to evaluate the diagnostic roles of echocardiography and cardiac magnetic resonance imaging (CMR) from Seoul, Korea and Rochester, NY, USA. Among them, patients who showed preserved LVEF (LVEF ≥50%) on echocardiography and who met the diagnostic criteria of HF with normal LV ejection fraction (HFNEF) according to 2007 European society of cardiology (ESC)‘s guideline were consecutively included in HFpEF group: (1) symptom or signs of HF, (2) Normal or mildly reduced LV systolic function, (3) evidence of abnormal LV relaxation, filling, diastolic distensiblity, and diastolic stiffness [6]. Symptoms and signs of HF were determined according to the modified Framingham criteria for the diagnosis of HF [7, 8].

Patients were excluded as following criteria: (1) patients who have significant valvular heart disease, significant heart block, acute coronary syndrome within 6 months or known cardiomyopathy causing diastolic HF, (2) hemodynamically unstable patients, (3) patients who are impossible to perform any of the tests: such as renal failure (estimated glomerular filtration rate < 30 mL/min), claustrophobia, presence of pacemaker, implantable cardiac defibrillator or metallic implant, pregnancy, malignancy.

Patients with hypertension without LV systolic dysfunction were enrolled in the hypertension group and normal subjects matched to the HFpEF group for age were included in normal group. Institutional review board at each institute approved the study protocol (IRB file number: 2008–08-079). Informed consent was confirmed by the IRB. Data were anonymized and analyzed independently by core lab in Samsung Medical Center.

### Data collection

#### 2D echocardiography and speckle tracking echocardiography

Comprehensive transthoracic echocardiography (M-mode, 2-D, and Doppler) was performed using commercially available equipment (Vivid 7, GE Medical system, Milwaukee, WI or Acuson 512, Siemens Medical Solutions, Mountain View, CA or Sonos 5500, Philips Medical System, Andover, MA, USA).

LV chamber size and wall thickness were measured by using 2005 American Society of Echocardiography (ASE)‘s guideline and standards [9]. LV mass was calculated using the conventional cube formula and LV hypertrophy (LVH) was determined according to the ASE’s chamber quantitation guideline (> 95 g/m^2^ for women, > 115 g/m^2^ for men) [9].

**Fig. 1:**
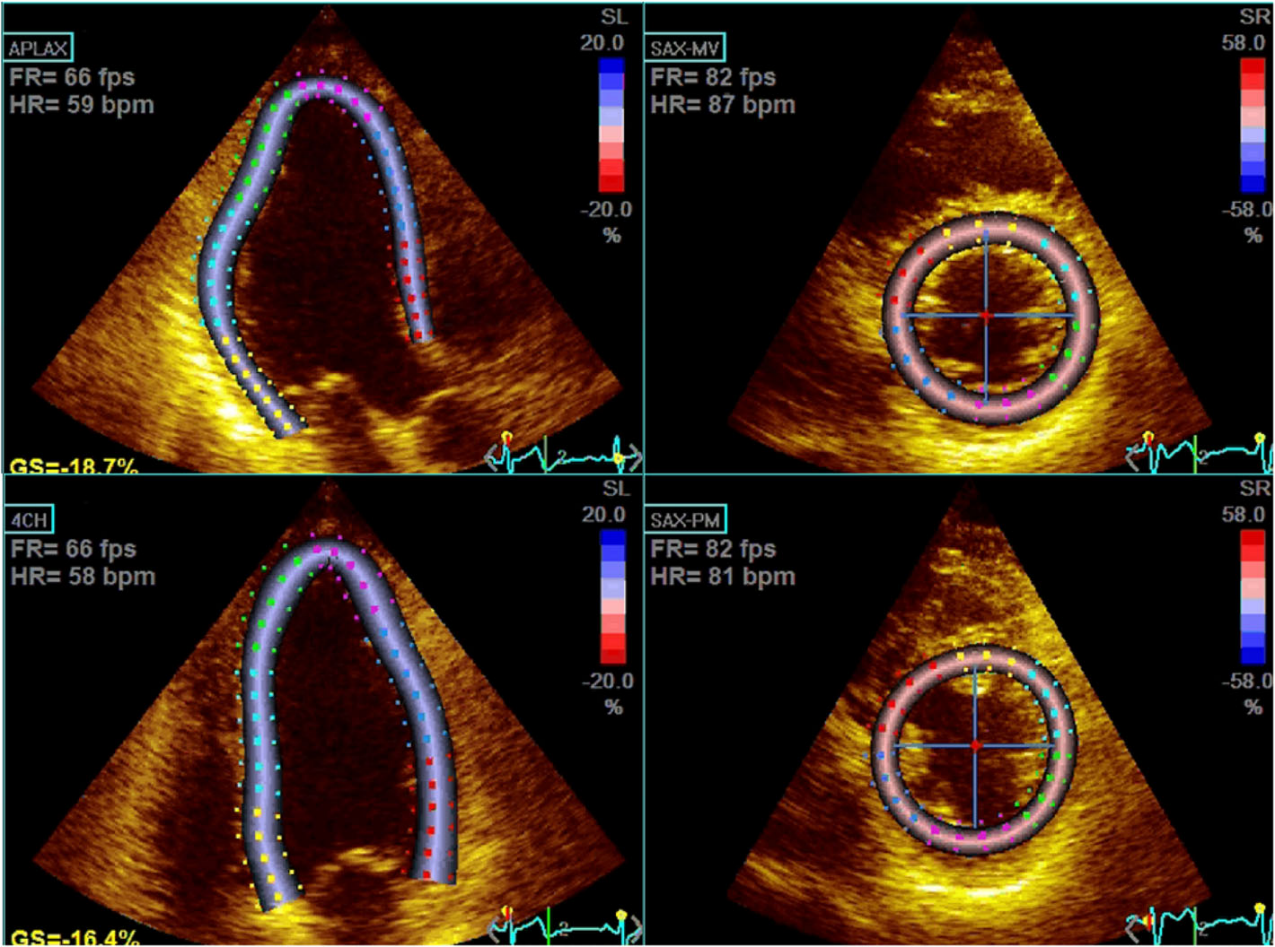
Example illustration of speckle tracking analysis. At the end-systolic phase, endocardial and epicardial borders were manually selected on each short axis and long axis view. Myocardial segment is consist of six circumferential regions (anterior, anteroseptal, inferoseptal, inferior, infeolateral, anterolateral) and three longitudinal regions (basal, mid, and apical)

Analysis of the 2D STE images was performed with a software package (EchoPAC, GE Ultrasound, Haifa, Israel). Loops of three consecutive cardiac cycles for 2D STE images were obtained. Two-dimensional data were analyzed using EchoPAC version 113.0.4 (GE Vingmed Ultrasound AS, Horten, Norway) by an experienced investigator blinded to all clinical information of the enrolled patients. Speckle-tracking analysis was performed using dedicated wall motion tracking software: Automated Function Imaging for 2D imaging (from GE Vingmed Ultrasound AS, Horten, Norway). 17 out of 152 values regarding regional longitudinal strain with poor-quality tracking or that provided aberrant curves despite manual adjustment were removed from analysis. (Fig. 1) Peak longitudinal, circumferential and radial strain were computed automatically for each LV segments and averaged value were reported as global strain. Early diastolic strain rate were assessed with the same manner. 16-segment model was applied in LV strain analysis because endocardial excursion and thickening of the apical cap are imperceptible.

### Statistical analysis

Data were analyzed using SPSS statistical software (version 20.0 for windows, SPSS, Inc., Chicago, IL). Categorical variables were presented as frequencies and percentages. The chi-square test or Fisher’s exact test was performed appropriately to test the difference of categorical variables between three groups. Continuous variables were presented as mean ± standard deviations. Oneway analysis of variances was performed to test the difference of continuous variables between three groups. Correlation between variables was assessed by Pearson’s method. Performance of each global strain for the prediction of HFpEF was evaluated by receiver-operating characteristics (ROC) analyses. Optimal cutoffs were calculated by DeLong’s method. Sensitivity, specificity, positive predictive value (PPV), and negative predictive value (NPV) were presented with proportions and 95% confidence intervals. *P* values < 0.05 were considered as significant.

## Results

### Study population and baseline clinical characteristics

**Fig. 2:**
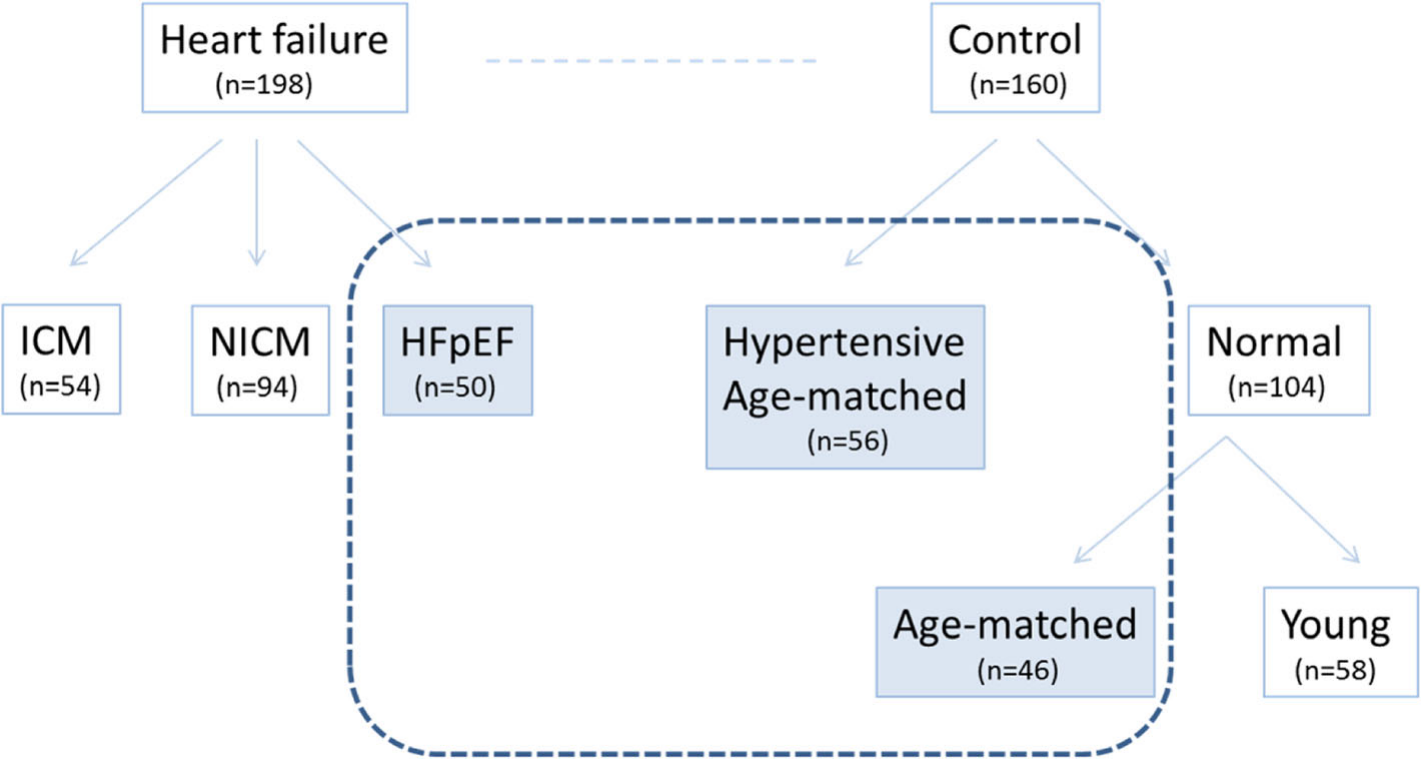
Study flow. HFpEF, heart failure with preserved ejection fraction, ICM, ischemic cardiomyopathy; NICM, non-ischemic cardiomyopathy

Among 258 patients enrolled in the IMAGING-HF study, there were 50 patients included in HFpEF group, 56 patients in hypertension group and 46 in age matched control group (Fig. 2). The median age at enrollment was 61 years (range, 30 years to 85 years) and consisted of 70 men (46.1%) and 82 women (53.9%). The baseline characteristics of the each group were described in detail in Table 1. Patients with HFpEF were older (67.4 ± 8.8 vs 59.8 ± 9.4 and 58.2 ± 6.3, *p* <  0.001) and tended to be obese compared to other groups. In HFpEF group, 70% of patients had hypertension and 84% of patients had atrial fibrillation previously. In addition, the mean N-terminal prohormone of brain natriuretic peptide (NT-proBNP) level was significantly higher (*p* = 0.021) and the rates of therapy with diuretics, angiotensin-converting enzyme inhibitor or angiotensin-receptor blocker and beta blocker were also higher in this group (*p* <  0.001, for all).

**Table 1:** Baseline clinical characteristics

	HFpEF (*n* = 50)	HTN (*n* = 56)	Age-matched control (*n* = 46)	*P* value
	Mean ± SD or n (%)			
Age (years)	67.4 ± 8.75	59.8 ± 9.43	58.2 ± 6.34	< 0.001*
Male sex	19 (38.0)	29 (51.8)	22 (47.8)	0.349
Body Mass Index (kg/m^2^)	31.5 ± 6.79	26.9 ± 3.67	24.8 ± 3.38	< 0.001*
Systolic BP (mmHg)	120.8 ± 25.0	136.6 ± 16.5	129.2 ± 15.1	0.003*
Diastolic BP (mmHg)	65.5 ± 20.1	83.7 ± 10.7	82.4 ± 10.6	< 0.001*
Heart rate (beat/min)	67.4 ± 13.0	69.3 ± 10.2	69.4 ± 10.7	0.626
Atrial fibrillation	42 (84.0)	5 (8.9)	1 (2.1)	< 0.001 *
Risk factors				
Hypertension	35 (70.0)	56 (100.0)	1 (2.2)	< 0.001*
Smoking				0.153
Ex-smoker	11 (22.0)	9 (16.1)	7 (15.2)	
Current smoker	6 (12.0)	12 (21.4)	7 (15.2)	
Hyperlipidemia	31 (62.0)	19 (33.9)	3 (6.5)	< 0.001*
Diabetes mellitus	16 (32.0)	1 (1.8)	0 (0.0)	< 0.001*
Prior myocardial infarction	4 (8.0)	0 (0.0)	0 (0.0)	< 0.001*
NT-proBNP (pg/dl)	677.6 ± 1077.5	87.7 ± 70.4	88.8 ± 102.9	0.021*
NYHA classification				< 0.001*
I	1 (100)	0 (0.0)	0 (0.0)	
II	18 (100)	0 (0.0)	0 (0.0)	
III	19 (100)	0 (0.0)	0 (0.0)	
Medications				
ACEi or ARB	22 (44.0)	8 (14.3)	0 (0.0)	< 0.001*
Beta-blocker	27 (54.0)	17 (30.4)	2 (4.3)	< 0.001*
Diuretics	28 (56.0)	20 (35.7)	0 (0.0)	< 0.001*
Calcium channel blocker	14 (28.0)	16 (28.6)	0 (0.0)	< 0.001*
Aspirin	30 (60.0)	15 (26.8)	1 (2.2)	< 0.001*
Statin	24 (48.0)	15 (26.8)	1 (2.2)	< 0.001*

The *p*-value denotes statistical significance comparing HFpEF, HTN and age-matched controls. *P <  0.05 by ANOVA (analysis of variance) or χ2-test. Data are listed as numbers (percentage of group), mean value. ACEi, angiotensin-converting-enzyme inhibitor; ARB, Angiotensin receptor blocker; BP, blood pressure; HFpEF, heart failure with preserved ejection fraction; HTN, hypertension; NT-proBNP, N-terminal prohormone of brain natriuretic peptide; NYHA, new york heart association; SD, standard deviation

### Echocardiographic findings and speckle tracking echocardiographic parameters

Echocardiographic parameters demonstrated normal-sized ventricle, normal wall thickness, and normal LVEF. The echocardiographic findings are summarized in Table 2. LV volume, EF, stroke volume and cardiac output were not statistically different among groups. However, indexed LV mass (LVMI) and indexed LA volume (LAVI) were significantly higher in the HFpEF and hypertension group. LVMI and LAVI were highest in HFpEF and lowest in normal subject (90.7 ± 29.6 vs 83.8 ± 20.2 vs 75.6 ± 19.4, *p* = 0.011; 38.9 ± 13.7 vs 32.6 ± 7.9 vs 30.9 ± 6.73, *p* <  0.001, respectively). By definition, LVH was found 12 in HFpEF group (24%), 8 in hypertension group (14.3%) and 2 in normal group (4.3%) (*p* = 0.010). In addition, mean septal E/e’ was highest in HFpEF group (14.30 ± 6.37), followed by hypertension group (9.82 ± 2.93) and normal group (8.51 ± 2.57) in decreasing order (*p* <  0.001).

**Table 2:** Baseline Echocardiographic data and Speckle Tracking Echocardiographic parameters

	HFpEF (*n* = 50)	HTN (*n* = 56)	Age-matched control (*n* = 46)	*P* value
	Mean ± SD or n (%)			
Echocardiographic parameter				
LVEDD (mm)	48.2 ± 5.04	48.6 ± 4.21	47.6 ± 3.39	0.456
LVESD (mm)	28.8 ± 3.98	28.4 ± 3.52	28.5 ± 2.72	0.889
IVSd (mm)	10.2 ± 2.71	9.11 ± 1.58	8.22 ± 1.28	< 0.001*
LVPWd (mm)	9.97 ± 2.14	8.62 ± 1.49	8.04 ± 1.38	< 0.001*
LV MI (g/m^2^, mean ± SEM)	90.7 ± 29.6	83.8 ± 20.2	75.6 ± 19.4	0.011*
Relative wall thickness	0.42 ± 0.11	0.36 ± 0.07	0.34 ± 0.06	< 0.001*
LVH by ASE guideline	12 (24.0)	8 (14.3)	2 (4.3)	0.010 *
LVEDV (ml)	101.0 ± 31.9	106.9 ± 25.9	107.9 ± 19.5	0.408
LVESV (ml)	35.7 ± 13.2	39.0 ± 11.7	38.9 ± 12.9	0.328
LAVI by area-length method (ml/m^2^)	38.9 ± 13.7	32.6 ± 7.89	30.9 ± 6.73	< 0.001*
LVEF (%)	64.9 ± 5.85	63.8 ± 5.11	64.0 ± 4.23	0.554
Stroke volume (ml)	85.3 ± 26.3	77.4 ± 16.5	72.9 ± 14.2	0.009 *
Cardiac output (L/min)	5.57 ± 1.7	5.28 ± 1.2	5.13 ± 1.2	0.298
Cardiac index (L/min/m^2^)	2.86 ± 0.7	2.95 ± 0.6	3.07 ± 0.8	0.344
E (m/sec)	0.83 ± 0.23	0.60 ± 0.16	0.62 ± 0.16	< 0.001*
A (m/sec)	0.75 ± 0.28	0.73 ± 0.16	0.67 ± 0.17	0.214
Septal e’(m/sec)	0.064 ± 0.021	0.063 ± 0.015	0.075 ± 0.018	0.002*
E/e’	14.30 ± 6.37	9.82 ± 2.93	8.51 ± 2.57	< 0.001*
Deceleration time	231.1 ± 54.9	240.8 ± 41.5	234.13 ± 44.5	0.572
Speckle Tracking Echocardiographic parameter				
Global longitudinal strain value				
Strain, %	−15.52 ± 5.32	−17.75 ± 3.12	−19.88 ± 2.04	< 0.001*
Strain rate, 1/s	0.97 ± 0.35	1.35 ± 0.29	1.60 ± 0.25	< 0.001*
Global circumferential strain value				
Strain, %	- 9.27 ± 2.19	−19.30 ± 3.14	−20.53 ± 3.30	< 0.001*
Strain rate, 1/s	1.03 ± 0.25	1.75 ± 0.42	1.94 ± 0.46	< 0.001*
Global radial strain value				
Strain, %	17.67 ± 8.22	38.39 ± 12.43	43.63 ± 11.92	< 0.001*
Strain rate, 1/s	−1.24 ± 0.48	− 1.85 ± 0.51	− 1.89 ± 0.39	< 0.001*

The *p*-value denotes statistical significance comparing HFpEF, HTN and age-matched controls. *P <  0.05 by ANOVA (analysis of variance) or χ2-test. Data are listed as numbers (percentage of group), mean value. ASE, American society of echocardiography; EDD, end-diastole dimension; EDV, end-diastolic volume; EF, ejection fraction; ESD, end-systolic dimension; ESV, end-systolic volume; HFpEF, heart failure with preserved ejection fraction; HTN, hypertension; IVSd, interventricular septum thickness at end-diastole; LAVI, Left atrial volume index; LV, left ventricular; LVH, LV hypertrophy; PWd, posterior wall thickness at end-diastole; SD, standard deviation.; SEM, standard error of the mean

**Fig. 3:**
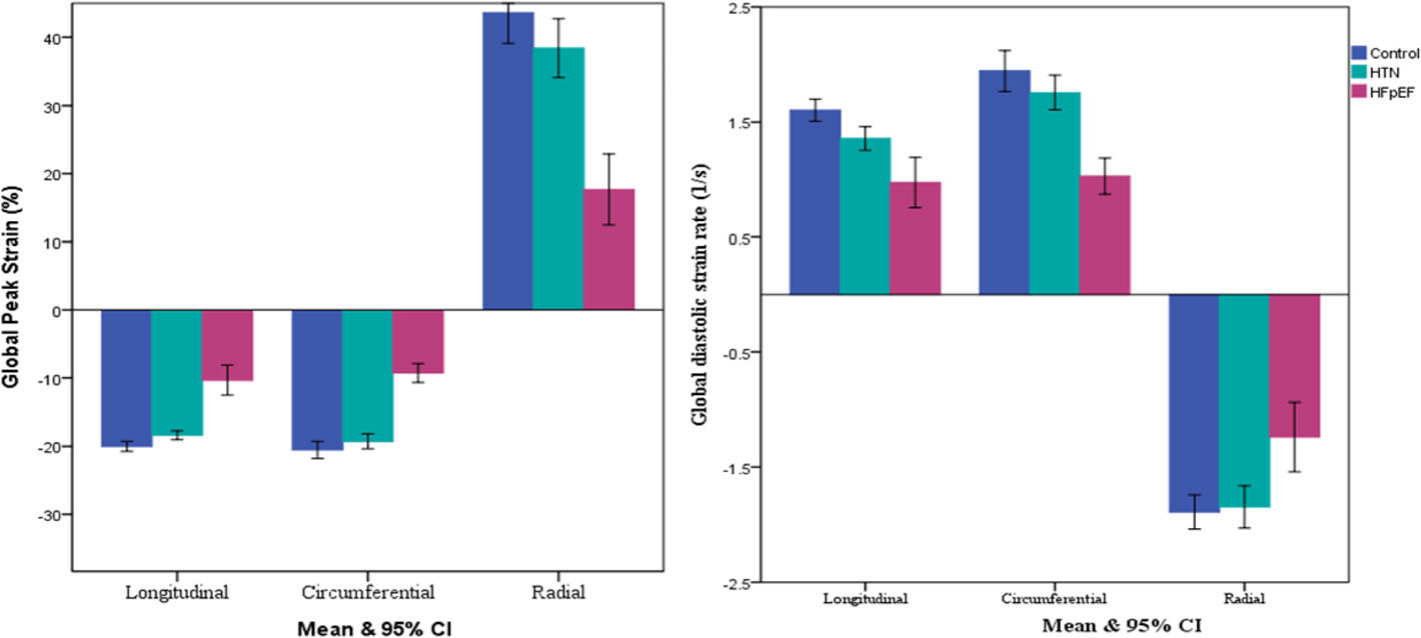
Distribution of global strain and strain rate among subgroups. CI, confidence interval; HFpEF, heart failure with preserved ejection fraction

**Fig. 4:**
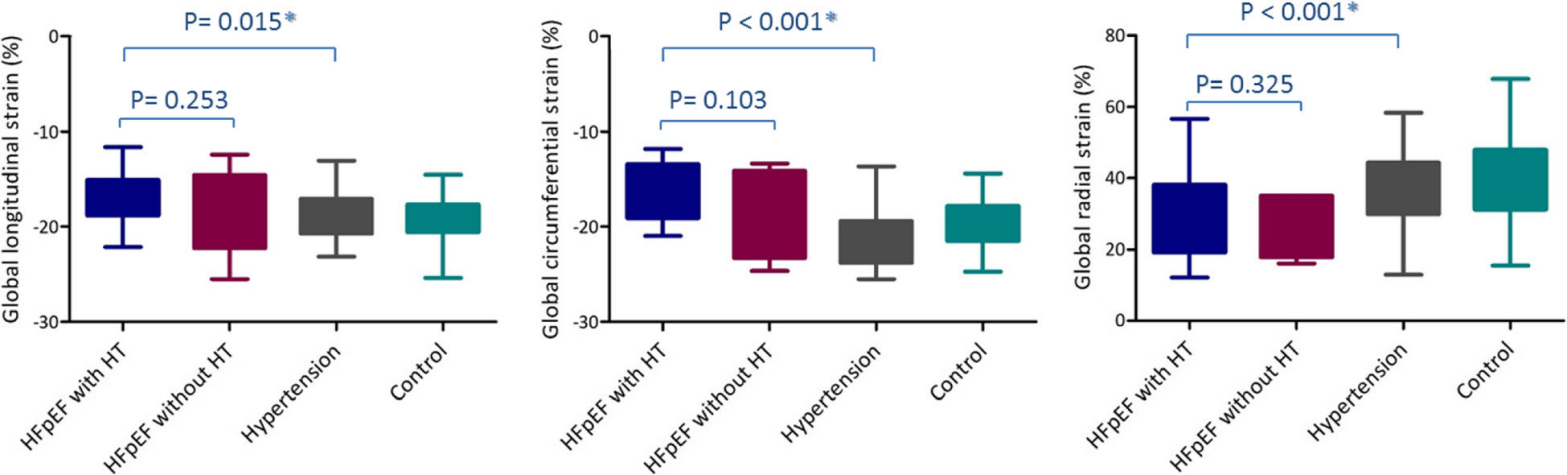
Comparison of global peak strain analysis among subgroups

In 2D STE, the HFpEF group showed marked reduction of global longitudinal, circumferential and radial strain and strain rate compared to the hypertension group or age-matched control group (*p* <  0.001, for all) (Table 2, Fig. 3). And these findings were consistently observed whether patients have hypertension or not (Fig. 4). In addition, reduction of regional longitudinal, circumferential and radial strain was evenly observed in the HFpEF group, compared to the hypertension group and control group in almost entire segment except basal and mid inferior-lateral segment (Table 3). Absolute difference of global and regional longitudinal, circumferential and radial peak strain and strain rate are shown in Table 4 and Table 5.

**Table 3:** Distribution of regional peak longitudinal, circumferential and radial strain

	Basal (Mean ± SD)			P-value	Mid (Mean ± SD)			*P*-value	Apical (Mean ± SD)			*P*-value
	HFpEF	HTN	Age-matched control		HFpEF	HTN	Age-matched control		HFpEF	HTN	Age-matched control	
Longitudinal												
Anterior	−13.0 ± 6.3	− 17.9 ± 5.5	−20.9 ± 4.2	< 0.001	− 14.8 ± 5.2	− 16.1 ± 7.2	− 20.0 ± 3.1	< 0.001	− 16.4 ± 7.9	− 16.8 ± 5.5	−20.6 ± 4.7	< 0.001
Ant.septal	− 13.9 ± 6.2	−16.4 ± 4.2	−17.5 ± 3.7	0.003	−16.6 ± 6.7	−19.1 ± 4.2	−21.2 ± 2.9	< 0.001	− 16.6 ± 7.9	− 19.3 ± 5.9	−22.2 ± 4.9	0.001
Inf.septal	−13.6 ± 6.2	−16.9 ± 3.8	−18.2 ± 3.5	< 0.001	−15.9 ± 7.1	− 18.5 ± 3.7	−19.9 ± 3.2	0.001				
Inferior	−16.9 ± 6.3	−19.1 ± 5.3	−21.1 ± 3.8	0.002	−16.2 ± 6.6	−18.9 ± 4.5	−20.4 ± 3.1	0.001	−17.1 ± 6.8	−19.8 ± 5.2	−23.9 ± 3.9	< 0.001
Inf.lateral	−15.6 ± 8.2	−18.1 ± 6.0	−19.2 ± 4.4	0.038	−13.5 ± 6.4	−17.5 ± 3.9	−18.3 ± 3.9	< 0.001	−15.6 ± 6.1	−17.1 ± 4.3	−20.5 ± 4.2	< 0.001
Ant.lateral	−14.0 ± 7.8	−17.5 ± 4.3	−19.1 ± 4.1	< 0.001	−14.2 ± 5.5	−16.3 ± 3.9	−18.9 ± 3.1	< 0.001				
Circumferential												
Anterior	−14.4 ± 7.8	−21.3 ± 6.0	−21.4 ± 3.8	0.001	−8.1 ± 4.7	−20.6 ± 6.9	−21.8 ± 5.1	< 0.001	−11.9 ± 6.4	−23.2 ± 7.0	−25.8 ± 5.6	< 0.001
Ant.septal	−12.2 ± 5.6	−26.2 ± 7.2	−26.8 ± 4.5	< 0.001	−8.2 ± 5.7	−24.9 ± 7.5	− 26.9 ± 5.1	< 0.001	−10.8 ± 8.7	− 24.7 ± 5.2	−27.3 ± 5.1	< 0.001
Inf.septal	−12.1 ± 5.7	−26.2 ± 6.7	− 26.7 ± 4.5	< 0.001	−9.5 ± 4.7	− 26.4 ± 6.2	−27.6 ± 4.3	< 0.001				
Inferior	−11.9 ± 7.7	−16.1 ± 6.1	−18.7 ± 6.7	0.009	−10.0 ± 5.3	−17.7 ± 5.4	− 18.6 ± 5.2	< 0.001	−7.9 ± 3.4	−23.2 ± 6.7	−25.3 ± 5.6	< 0.001
Inf.lateral	−9.9 ± 5.9	−10.7 ± 8.4	−11.3 ± 7.2	0.853†	−8.6 ± 6.5	−11.6 ± 6.4	−12.2 ± 7.8	0.527†	−10.0 ± 4.1	−21.1 ± 6.2	−22.7 ± 6.1	< 0.001
Ant.lateral	−7.3 ± 3.7	−11.1 ± 5.8	−11.4 ± 6.0	0.073	−9.2 ± 5.6	−13.3 ± 5.8	−13.7 ± 5.3	0.042				
Radial												
Anterior	22.0 ± 15.3	42.0 ± 15.9	49.1 ± 15.6	< 0.001	14.7 ± 12.5	40.5 ± 15.6	46.4 ± 22.2	< 0.001	10.5 ± 7.7	23.6 ± 15.2	25.4 ± 13.9	0.005
Ant.septal	16.1 ± 12.9	35.8 ± 12.9	43.4 ± 13.8	< 0.001	12.8 ± 12.7	38.1 ± 13.6	43.2 ± 17.5	< 0.001	8.2 ± 7.4	25.3 ± 14.8	28.3 ± 14.3	< 0.001
Inf.septal	15.5 ± 8.9	43.4 ± 19.1	48.9 ± 15.6	< 0.001	18.8 ± 20.3	46.7 ± 17.0	47.9 ± 16.7	< 0.001				
Inferior	20.7 ± 11.5	50.4 ± 23.6	61.9 ± 18.3	< 0.001	21.7 ± 18.5	50.2 ± 17.7	51.4 ± 19.9	< 0.001	7.6 ± 5.5	23.2 ± 14.2	27.7 ± 15.8	< 0.001
Inf.lateral	27.5 ± 14.2	55.8 ± 23.3	65.3 ± 19.8	< 0.001	21.6 ± 16.3	50.8 ± 18.2	55.2 ± 21.8	< 0.001	10.1 ± 7.4	22.4 ± 15.3	24.9 ± 15.3	0.008
Ant.lateral	28.2 ± 15.2	49.9 ± 21.5	59.0 ± 18.0	< 0.001	19.1 ± 15.6	47.2 ± 17.2	53.2 ± 22.9	< 0.001				

The p-value denotes statistical significance comparing HFpEF, HTN and age-matched controls. †*P* > 0.05 by ANOVA (analysis of variance) or χ2-test. Data are listed as mean value. Ant, anterior; Ant.lat, anterolateral; Ant.septal, anteroseptal; HFpEF, heart failure with preserved ejection fraction; HTN, hypertension; Inf, inferior; Inf.lat, infeolateral; Inf.septal, inferoseptal; SD, standard deviation

**Table 4:** Absolute and percent (%) difference of Global systolic strain and strain rate between groups

	HFpEF and control	HTN and control	HFpEF and HTN
Global longitudinal strain value			
Strain, %	4.36 (21.9)	2.13 (10.7)	2.23 (12.6)
Strain rate, 1/s	0.63 (39.4)	0.91 (46.9)	0.65 (34.4)
Global circumferential strain value			
Strain, %	11.3 (54.8)	1.23 (6.00)	10.0 (52.0)
Strain rate, 1/s	0.25 (15.6)	0.19 (9.79)	0.04 (2.11)
Global radial strain value			
Strain, %	25.9 (59.5)	5.24 (12.0)	20.7 (54.0)
Strain rate, 1/s	0.38 (28.1)	0.72 (41.1)	0.50 (27.0)

HFpEF, heart failure with preserved ejection fraction; HTN, hypertension

**Table 5:** Absolute and percent (%) difference of Regional Strain between groups

	Basal		Mid		Apical	
	HFpEF - control	HTN - control	HFpEF - control	HTN - control	HFpEF - control	HTN - control
Longitudinal						
Anterior	7.9 (37.7)	3.0 (14.4)	5.2 (26.0)	3.9 (19.5)	4.2 (20.3)	3.8 (18.4)
Ant.septal	3.6 (20.5)	1.1 (6.28)	4.6 (21.6)	2.1 (9.90)	5.6 (25.2)	2.9 (13.1)
Inf.septal	4.6 (25.3)	1.3 (7.14)	4.0 (20.1)	1.4 (7.01)		
Inferior	4.2 (19.9)	2.0 (9.47)	4.2 (20.5)	1.4 (6.86)	6.8 (28.4)	4.1 (17.2)
Inf.lateral	4.8 (26.8)	1.1 (5.73)	4.8 (26.2)	0.8 (4.37)	4.9 (23.9)	3.4 (16.6)
Ant.lateral	5.1 (26.7)	16 (8.37)	4.7 (24.8)	2.6 (13.8)		
Circumferential						
Anterior	7.0 (32.7)	0.1 (0.4)	13.7 (62.8)	1.2 (5.53)	13.9 (53.9)	2.6 (10.1)
Ant.septal	14.6 (54.5)	0.6 (2.23)	16.7 (67.1)	2.0 (7.43)	17.4 (68.8)	2.6 (9.51)
Inf.septal	14.6 (54.7)	0.5 (1.87)	18.1 (65.6)	1.2 (4.34)		
Inferior	6.8 (36.4)	2.6 (1.39)	8.6 (46.2)	0.9 (4.83)	17.4 (68.8)	2.1 (8.34)
Inf.lateral	1.4 (12.4)	0.6 (5.31)	3.0 (25.8)	0.6 (4.91)	12.7 (56.0)	1.6 (7.04)
Ant.lateral	4.1 (36.0)	0.3 (2.62)	4.5 (32.8)	0.4 (2.91)		
Radial						
Anterior	27.1 (55.2)	7.1 (14.5)	31.7 (68.3)	5.9 (12.7)	14.9 (58.7)	1.8 (7.08)
Ant.septal	27.3 (62.9)	7.6 (17.5)	30.4 (70.4)	5.1 (11.8)	20.1 (71.0)	3.0 (10.6)
Inf.septal	33.4 (76.9)	5.5 (12.6)	29.1 (60.8)	11.2 (2.52)		
Inferior	41.2 (66.6)	11.5 (18.6)	29.7 (57.8)	1.2 (2.33)	20.1 (72.6)	4.5 (16.2)
Inf.lateral	37.8 (57.9)	9.5 (14.5)	33.6 (60.9)	4.4 (7.97)	14.8 (59.4)	2.5 (10.0)
Ant.lateral	30.8 (52.2)	9.1 (15.4)	34.1 (64.1)	6.0 (11.2)		

Ant, anterior; Ant.lat, anterolateral; Ant.septal, anteroseptal; HFpEF, heart failure with preserved ejection fraction; HTN, hypertension; Inf, inferior; Inf.lat, infeolateral; Inf.septal, inferoseptal

**Fig. 5:**
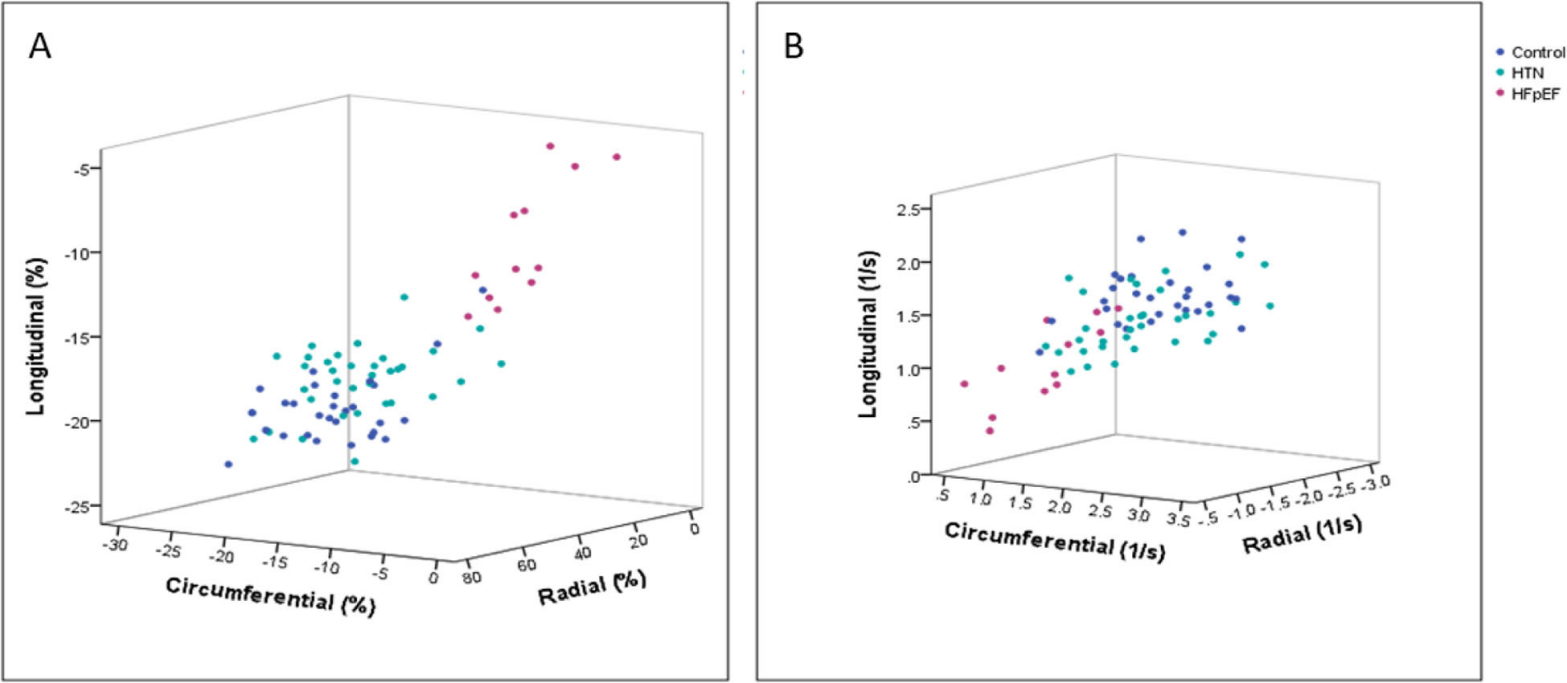
Correlation between two global strain (A) and strain rate (B). Longitudinal, circumferential and radial strain and strain rate were significantly correlated with each other (Longitudinal and circumferential, *r* = 0.78, *p* < 0.001; longitudinal and radial, *r* = 0.66, p < 0.001; radial and circumferential, *r* = 0.70, *p* < 0.001)

Interestingly, these global strains showed significant correlation between each other (longitudinal and circumferential, *r* = 0.78, *p* <  0.001; longitudinal and radial, *r* = 0.66, *p* <  0.001; radial and circumferential, *r* = 0.70, *p* <  0.001) (Fig. 5).

### Association with LV diastolic function

Reduced global peak longitudinal strain was associated with dilated LA cavity size (Pearson correlation *r* = 0.35, *p* <  0.001). Further in detail, the averaged basal peak longitudinal strain was weakly correlated with E/e’ (*r* = 0.20, *p* = 0.024). However, there was no significant correlation between E/E’ and any of global longitudinal, circumferential and radial strain.

### Diagnostic performance of global longitudinal, circumferential and radial strain

**Fig. 6:**
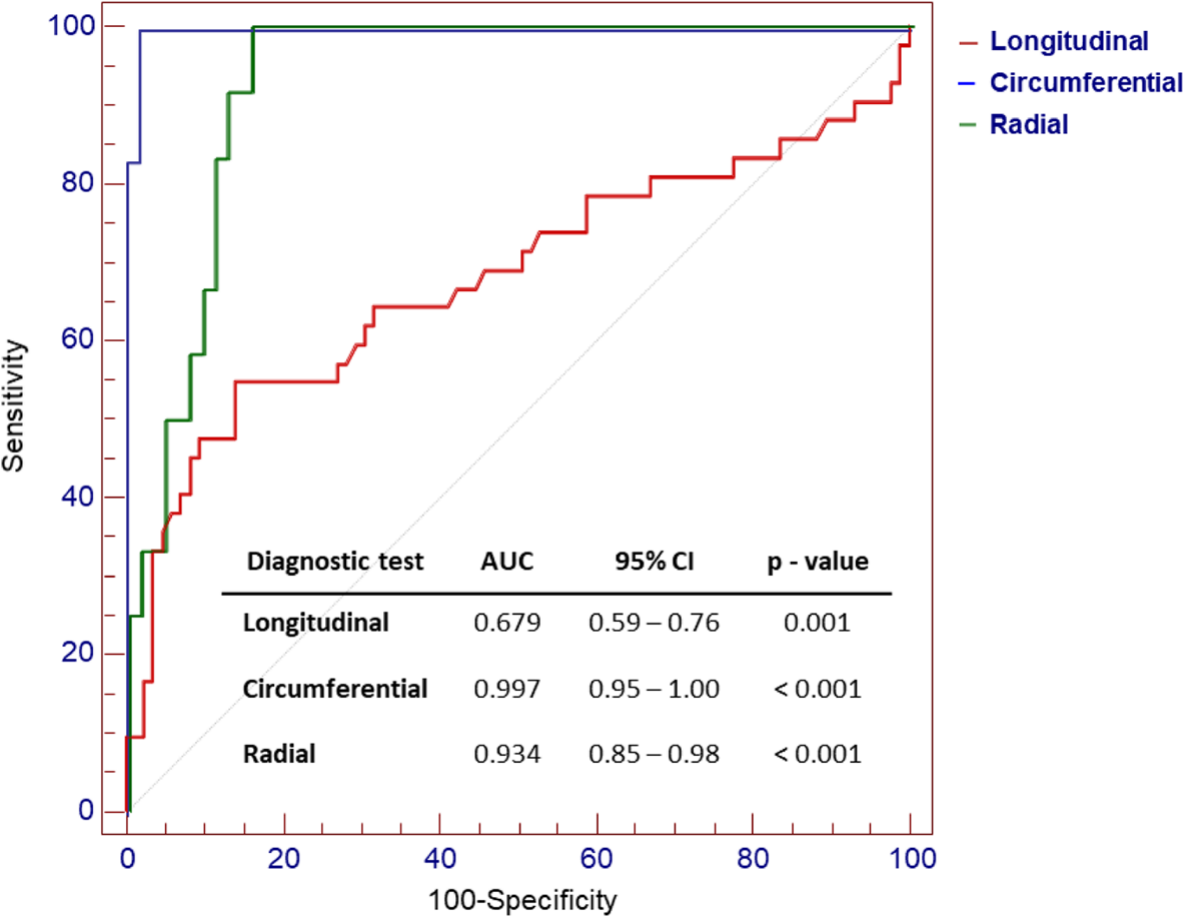
Predictive performance of global peak strain (%) for the diagnosis of HFpEF. AUC, area under the curve; CI, confidence interval

In the ROC curve analysis, global longitudinal, circumferential and radial peak strain failed to predict the hypertension. However, the diagnostic performance of global circumferential and radial strain to predict the HFpEF were excellent, though that of global longitudinal strain (GLS) was fair (area under the curve (AUC) = 0.99, *P* <  0.001; AUC = 0.93, *P* <  0.001; AUC = 0.68, *p* = 0.001; respectively) (Fig. 6). Meanwhile, the diagnostic performance of NT-proBNP to predict the HFpEF was fair (AUC = 0.83, *p* <  0.001). There was no significant correlation between NTproBNP and each of SL, SC or SR.

The optimal cut-off of GLS was − 16.7 (%), with sensitivity = 54.8% (95% CI = 38.7–70.2), specificity = 85.9% (76.6–92.5), PPV = 65.7% (47.8–80.9), NPV = 79.3% (69.6–87.1). The optimal cut-off of global circumferential strain (GCS) was − 13.8 (%), with 100% of sensitivity, 98.4% of specificity, 92.3% of PPV and 100% of NPV (*p* <  0.001) and the optimal cut-off global radial strain (GRS) was 29.1(%), with 100% of sensitivity, 84.1% of specificity, 54.5% of PPV and 100% of NPV (*p* <  0.001).

## Discussion

In this multicenter prospective study, we investigated global and regional LV strains using 2D STE in patients with HFpEF and compared those parameters with that of patients with hypertension and normal subjects.

The main findings of this study are (1) impaired global longitudinal, circumferential and radial peak strain and strain rate were observed in HFpEF patients compared to the hypertension patients and normal subjects in decreasing order; (2) regional longitudinal, circumferential and radial strain were also homogeneously reduced in most region; (3) decreased basal longitudinal strains were related to increased E/E’, while reduced GLS was significantly associated to LA enlargement; (4) the diagnostic performance of GLS to predict the HFpEF was fair with the best cut-off value of − 16.7 (%); (5) the diagnostic performance of GCS to predict the HFpEF was excellent with the best cut-off value of − 13.8 (%), respectively.

GLS is considered to be a reliable predictor of HFpEF [7, 10, 11]. It is challenging to detect subclinical cardiac dysfunction using conventional echocardiography, 2D STE allows more precise evaluation of the cardiac function and mechanics in HFpEF. In the majority of studies using 2D STE, GLS was significantly lower in HFpEF patients. GLS predominantly detects longitudinal movement of LV. Considering that LV motion is complex and dynamic with multidirectional contraction and relaxation of each layers of myocardial fibers, GCS should be reduced in the same manner. Apparently, GCS was significantly lower in HFpEF in previous studies [2, 12]. In our study, GCS as well as GLS reduced in HFpEF and the predictive performance of GCS was even greater than GLS for the diagnosis of HFpEF. Further, all GLS, GCS and GRS were significantly associated together, which suggests that the multidirectional concurrent movement of each layered myocardial fiber can be approached comprehensively by GCS and GRS together with GLS. However in some studies, GCS and GRS failed to demonstrate significant difference between HFpEF and control [13, 14]. This inconsistency could be a result of technical limitation such as angle dependence, signal noise, intra-observer, inter-observer and inter-vendor variability or limited number of study focused on GCS and GRS.

Meanwhile, finest STE index could also be evaluated in order to give further detail [15]. According to the recent study, increased LV mechanical dispersion is associated with increased risk of cardiovascular disease including heart failure, ischemic heart disease and ventricular arrhythmias [16].

Previously, the HFpEF have been considered to be as same as diastolic dysfunction. Accordingly, the most important index in determining of HFpEF was conventional parameters of diastolic function; such as E, E/A ratio, e’, the E/e’ ratio and LA size [17]. Nevertheless, E/e’ was not a sensitive indicator for early detection of HFpEF [18, 19]. Earlier detection of diastolic dysfunction could be achieved through evaluation using GLS [2, 12].

In the current study, E/e’ showed better correlation with averaged basal peak longitudinal strain than with GLS. It indicates that E/e’ mainly represents local diastolic blood flow and perpendicular movement of mitral annular tissue rather than entire myocardial relaxation. Further accurate and precise assessment of diastolic dysfunction should encompass multi-directional myocardial deformation. Taking together, GCS and GRS should be considered as substantial predictors of HFpEF.

There are several strengths of the current study. According to the previous study which investigated STE in 219 HFpEF patients and compared to the hypertensive patients and normal controls, GLS and GCS were demonstrated to be lower in HFpEF population compared to hypertension or normal subject [2]. Meanwhile, 83% of the study population was white race in the study. As we know, this is the first study that investigated multi-directional STE in patients with HFpEF in Korean population. Results in the current study were similar to the previous report despite different ethnicity, which is one of the added values of our study.

Furthermore in the current study, GRS as well as GLS and GCS were investigated to assess sensitivity of radial strains to detect subclinical cardiac dysfunction. Although there remains controversy on the diagnostic performance of GRS, our result suggests that GRS could be useful in the diagnosis of HFpEF in the future if the technical i could be solved.

In addition, our results provide regional strains for each segment of LV. It is accepted that the regional cardiac performance changes earlier than the global function [20]. This could be another added value of the current study.

### Study limitations

This study has several limitations. There are significant differences in age among groups. Since HFpEF occurs more often in the elderly, the average age of patients included in the HFpEF group is inevitably higher. Unfortunately, this can lead to selection bias. Meanwhile, it is recognized that strain values vary with increasing age. According to the earlier study, reference limit was lower in the higher mean aged population [21]. However, the differences of strain values between 2 different age groups are less than 1.3 (range, 0.1 to 1.3), which does not seem to be significant. Authors concluded that age was not significantly associated with strain measures after multivariable adjustment for clinical. In our study, the differences in strain values for each group is greater, even considering that strain decreases with age, this differences could be influenced by the disease status.

Patients enrolled in this study may not be representative of HFpEF and hypertensive patients in the community, because of small sample size of patients and their racial differences. Moreover, large portion of patients with HFpEF had atrial fibrillation, strain values can be variable even if the averaged value of 5–7 measurements were used. Lastly, global longitudinal, circumferential, and radial strain and strain rate were evaluated only in one echocardiographic projection, it would be more accurate to assess them using 3D echocardiography. In the future, the prognostic value of HFpEF, including 2D STE and 3D echocardiographic parameters will be established in multicenter, larger population studies.

## Conclusions

In summary, peak global strain and regional strain were homogenously reduced in HFpEF patients compared to the hypertension patients and normal subjects in decreasing order. The predictive performance of GCS was greater than that of GLS or GRS in the diagnosis of HFpEF. Earlier information on the multi-directional LV deformation can provide early detection of diastolic dysfunction, which would improve clinicians understanding and management of HFpEF.

## Data Availability

The datasets during and/or analysed during the current study available from the corresponding author on reasonable request.
